# 

**Published:** 1852-06

**Authors:** 


SUBSCRIBERS ARE CAUTIONED

Not to pay money on account of this Journal to any person or persons except C. W. JAMES, ISRAEL E. JAMES, HENRY M. LEWIS, or their AUTHORISED Agents, as no other payments will be acknowledged by the Publishers.

SUBSCRIBERS are authorised to make remittances by mail at the risk of the Publishers when the Post Master's certificate is obtained.

LINDSAY & BLAKISTON, *Publishers*,
Philadelphia.

### NOTICE TO CORRESPONDENTS.

Communications and Books for notice should be addressed to the Editors, care of Messrs. Lindsay & Blakiston.

Letters, &c., connected with the *business affairs* of the Journal should be addressed to the Publishers.

Papers for publication must be received *before* the 20th of the month, or they cannot appear in the forthcoming number.

The following Journals have been received in exchange:

New York Medical Times.
Medical News. Philadelphia.
Buffalo Journal.
L'Union Medicale.
New York Medical Gazette.
The Boston Medical and Surgical Journal. (Weekly.)
Stethoscope and Virginia Medical Gazette.
Western Journal.
New Jersey Medical and Surgical Reporter.
Nordamericanischer Monatsbericht.
Transylvania Medical Journal.
Nashville Journal of Medicine and Surgery.
Southern Medical and Surgical Journal.
New Hampshire Journal of Medicine.
Opal, from the Utica Insane Asylum.
Dental Times and Advertiser.
The London Lancet. (Weekly, London.)
The Medical Times and Gazette. (Weekly, London.)
Dublin Medical Press.
London Journal of Medicine.
British and Foreign Medico-Chirurgical Review.
Edinburgh Monthly Journal.
Journal of Psychological Medicine.
Journal of Pharmacy.
North Western Medical Intelligencer.
American Journal of Insanity.

---

### BOOKS RECEIVED.

Report of the Ohio Lunatic Asylum for the year 1851.
Graham's Elements of Chemistry. From Blanchard & Lea.
Miller's Principles of Surgery.
Mendenhall's Vade Mecum.
Theses. From E. Brown-Séquard, M. D.
Wood's Practice of Medicine. New Edition. From Lippincott & Grambo.
Cooper's Lectures on Surgery. From Blanchard & Lea.
Sketches of Brazil, by Dr. Dundas. From the Author.
Bartlett on Fevers. New Edition. From Blanchard & Lea.
Beck on Infant Therapeutics.

---

Communications have been received from:—

E. D. Daily, M. D., Maryland.
D. H. Gibson, M. D., Choctaw Nation.
T. M. Jacks, Arkansas.

---

The foreign correspondents of the Examiner will please direct their Exchanges and other communications to the care of Mr. Thos. Delf, No. 12 Paternoster Row, London, or Mr. H. Bossange, 21 Bis, Quai Voltaire, Paris.

